# Carbon Nanotubes Modified by Venturello Complex as Highly Efficient Catalysts for Alkene and Thioethers Oxidation With Hydrogen Peroxide

**DOI:** 10.3389/fchem.2019.00858

**Published:** 2019-12-13

**Authors:** Vasiliy Yu Evtushok, Irina D. Ivanchikova, Olga Yu Podyacheva, Olga A. Stonkus, Arina N. Suboch, Yuri A. Chesalov, Olga V. Zalomaeva, Oxana A. Kholdeeva

**Affiliations:** ^1^Department of Fine Organic Synthesis and Renewable Energy Sources, Boreskov Institute of Catalysis, Novosibirsk, Russia; ^2^Department of Natural Sciences, Novosibirsk State University, Novosibirsk, Russia

**Keywords:** selective oxidation, heterogeneous catalysis, hydrogen peroxide, carbon nanotubes, Venturello complex

## Abstract

In this work, we elaborated heterogeneous catalysts on the basis of the Venturello complex [PO_4_{WO(O_2_)_2_}_4_]^3−^ (PW_4_) and nitrogen-free or nitrogen-doped carbon nanotubes (CNTs or N-CNTs) for epoxidation of alkenes and sulfoxidation of thioethers with aqueous hydrogen peroxide. Catalysts PW_4_/CNTs and PW_4_/N-CNTs (1.8 at. % N) containing 5–15 wt. % of PW_4_ and differing in acidity have been prepared and characterized by elemental analysis, N_2_ adsorption, IR spectroscopy, HR-TEM, and HAADF-STEM. Studies by STEM in HAADF mode revealed a quasi-molecular dispersion of PW_4_ on the surface of CNTs. The addition of acid during the immobilization is not obligatory to ensure site isolation and strong binding of PW_4_ on the surface of CNTs, but it allows one to increase the PW_4_ loading and affects both catalytic activity and product selectivity. Catalytic performance of the supported PW_4_ catalysts was evaluated in H_2_O_2_-based oxidation of two model substrates, cyclooctene and methyl phenyl sulfide, under mild conditions (25–50°C). The best results in terms of activity and selectivity were obtained using PW_4_ immobilized on N-free CNTs in acetonitrile or dimethyl carbonate as solvents. Catalysts PW_4_/CNTs can be applied for selective oxidation of a wide range of alkenes and thioethers provided a balance between activity and selectivity of the catalyst is tuned by a careful control of the amount of acid added during the immobilization of PW_4_. Selectivity, conversion, and turnover frequencies achieved in epoxidations over PW_4_/CNTs catalysts are close to those reported in the literature for homogeneous systems based on PW_4_. IR spectroscopy confirmed the retention of the Venturello structure after use in the catalytic reactions. The elaborated catalysts are stable to metal leaching, show a truly heterogeneous nature of the catalysis, can be easily recovered by filtration, regenerated by washing and evacuation, and then reused several times without loss of the catalytic performance.

## Introduction

Epoxides are widely used as valuable building blocks in both base and fine organic synthesis due to specific reactivity of the oxirane ring (Dusi et al., 2000; Sienel et al., 2000; Bauer et al., 2001; Sheldon and van Vliet, 2001; Adolfsson, 2004). Oxidation of alkenes by various peroxide-containing compounds is one of the main ways to obtain epoxides (Swern, 1971). Most synthetic approaches are based on the employment of peroxy acids (Prilezhaev reaction) (Swern, 1953, 1971) or alkyl hydroperoxides (Sheldon and Kochi, 1981) as oxidants. However, the use of dilute aqueous hydrogen peroxide (up to ~50 wt. %) as a green oxidizing agent is of considerable interest from both economic and environmental points of view (Strukul, 1992; Jones, 1999; Clerici and Kholdeeva, 2013). The selective epoxidation of alkenes with H_2_O_2_ can only be accomplished in the presence of catalysts that enable heterolytic activation of H_2_O_2_ (Sheldon and Kochi, 1981; Strukul, 1992; Clerici and Kholdeeva, 2013). Transition metal complexes often have high epoxidation activity and selectivity, and their properties are easy to regulate due to a wide variety of ligands (Jørgensen, 1989; Lane and Burgess, 2003; Hauser et al., 2013; Srour et al., 2013). Unfortunately, organic ligands are inherently unstable toward oxidation, which can lead to poor catalyst productivity and recyclability (Lane and Burgess, 2003).

The catalytic oxidation of thioethers is an important research field because sulfoxides and sulfones, the products of thioethers oxidation, are widely used as intermediates in the synthesis of fine chemicals and pharmaceuticals (Madesclaire, 1986; Fernandez and Khiar, 2003; Rostamnia and Mohsenzad, 2018; Li and Jiang, 2019). Moreover, liquid-phase oxidation of S-containing organic compounds is considered as a promising methodology for removal of S-containing compounds from fuels (Campos-Martin et al., 2010) and decontamination of chemical warfare agents (Dong et al., 2017).

Polyoxometalates (POM) are anionic transition metal-oxygen clusters widely known in catalysis and materials science for their unique features, such as thermodynamic stability to oxidation, stability to hydrolysis in a wide range of pH and thermal degradation, tunable composition, solubility, redox activity, and acidity (Pope, 1983; Hill, 1998; Kholdeeva et al., 2010; Weinstock, 2011; Cronin and Müller, 2012). Tetranuclear phosphotungstate [PO_4_{WO(O_2_)_2_}_4_]^3−^ (PW_4_), also known as Venturello complex, has long been used as an efficient homogeneous catalyst for H_2_O_2_-based epoxidations (Venturello et al., 1983, 1985, 1986). The preparation of PW_4_ is simple and affordable (Venturello et al., 1983). Importantly, it is not prone to solvolysis/hydrolysis in typical conditions of liquid-phase oxidation. In terms of catalytic characteristics, PW_4_ shows high selectivity and H_2_O_2_ utilization efficiency (Duncan et al., 1995). A range of homogeneous catalytic systems based on PW_4_, mostly biphasic, have been developed (Ishii et al., 1988; Oguchi et al., 1989; Sakaguchi et al., 1996; Sato et al., 1996; Sun et al., 2001; Zuwei et al., 2001; Lambert et al., 2003; Kaur and Kozhevnikov, 2004; Ding et al., 2008; Leclercq et al., 2012; Kamata et al., 2014). Quite often, commercial heteropolyacid H_3_PW_12_O_40_ (PW_12_) was used as a pre-catalyst and PW_4_ was generated *in situ* (Venturello et al., 1983; Ishii et al., 1988; Duncan et al., 1995).

Due to these evident advantages, PW_4_ has received significant attention as an active component for construction of heterogeneous epoxidation catalysts (Neumann and Miller, 1995; Neumann and Cohen, 1997; Hoegaerts et al., 2000; Sakamoto and Pac, 2000; Sels et al., 2000; Kovalchuk et al., 2007; Sofia et al., 2009; Maksimchuk et al., 2010; Leng et al., 2011; Doherty et al., 2012; Swalus et al., 2013; Nojima et al., 2015; Peng et al., 2016; Masteri-Farahani and Modarres, 2017; Shen et al., 2017; You et al., 2018). Silica modified by various cationic functional groups was widely used as support for immobilization of PW_4_ (Neumann and Miller, 1995; Neumann and Cohen, 1997; Hoegaerts et al., 2000; Sakamoto and Pac, 2000; Kovalchuk et al., 2007; Sofia et al., 2009). However, most of these catalysts turned out less active than homogeneous PW_4_ (Neumann and Miller, 1995; Neumann and Cohen, 1997; Hoegaerts et al., 2000; Kovalchuk et al., 2007). Some of the SiO_2_-supported catalysts were prone to leaching and/or had a poor recyclability (Hoegaerts et al., 2000; Kovalchuk et al., 2007) (sometimes information about reusability was not provided Neumann and Miller, 1995; Sakamoto and Pac, 2000). Several catalysts based on ion-exchange organic polymers and PW_4_ have been developed (Sels et al., 2000; Doherty et al., 2012; Swalus et al., 2013; Peng et al., 2016; Shen et al., 2017; You et al., 2018). In general, catalysts PW_4_/polymer showed high selectivity for epoxides and most of them could be recycled without a decrease in attainable conversion and selectivity (Sels et al., 2000; Swalus et al., 2013; Peng et al., 2016; Shen et al., 2017; You et al., 2018). However, their stability in terms of activity (retention of the reaction rate during the recycling) was not often addressed (Sels et al., 2000; Peng et al., 2016; Shen et al., 2017; You et al., 2018). PW_4_ and PW_12_ incorporated into the cages of the metal-organic framework MIL-101(Cr) demonstrated fairly good catalytic activity, selectivity, and recyclability (Maksimchuk et al., 2010). Mizuno and co-workers have developed supported catalysts PW_4_/Zn-SnO_2_, which appeared even more active than homogeneous PW_4_ and not prone to leaching (Nojima et al., 2015). However, activity of the PW_4_/Zn-SnO_2_ catalysts slightly decreased during their recycling. Recently, graphene oxide was used as a support for immobilization of peroxophosphotungstates (Masteri-Farahani and Modarres, 2017). PW_4_ supported on graphene oxide sheets exhibited unusually high turnover frequency (TOF = 500–1,000 h^−1^) values but the catalyst suffered from leaching. Thus, there is not yet a final solution of the problem and the development of an effective heterogeneous catalyst on the basis of PW_4_ that would be highly active, selective, and stable remains a challenging goal.

Carbon nanomaterials (CNMs), such as carbon nanotubes (CNTs) and carbon nanofibers have found applications as key components in electronic and energy materials, molecular sensors, and catalysis (Eder, 2010). Polyoxometalates supported on CNMs have been used mostly as electrocatalysts in fuel cells and water splitting systems (Pan et al., 2006; Toma et al., 2010; Cui et al., 2011; Kawasaki et al., 2011; Guo et al., 2013; Ji et al., 2015). Various techniques for immobilization of POMs on the CNMs surface were used, including adsorption from solution (Pan et al., 2006; Giusti et al., 2009; Kawasaki et al., 2011), impregnation (Salavati et al., 2010; Wang et al., 2010), electrostatic attachment to cationic functional groups (Toma et al., 2010), and covalent binding of organo-functionalized POMs (Chen et al., 2014; Ji et al., 2015). However, very few materials have been tested for selective oxidation reactions. In particular, Cs_2.5_H_0.5_PW_12_O_40_/CNTs prepared by impregnation technique was used for the oxidative desulfurization with a desulfurization efficiency up to 100%, but it showed a slight deterioration of the efficiency during recycling (Wang et al., 2010).

Doping of CNMs by nitrogen (N-CNMs) by a catalytic growth route leads to the incorporation of N atoms into the carbon matrix throughout the volume, including the surface layer (García-Bordejé et al., 2014). The formation of different surface N species may diversify approaches to immobilization of catalytically active species and affect specific surface properties of heterogeneous catalysts (García-Bordejé et al., 2014; Arrigo et al., 2015). Application of N-CNMs in catalysis was mostly directed to the preparation of supported catalysts with metal nanoparticles or single atoms (Arrigo et al., 2012; Ayusheev et al., 2014; Bulushev et al., 2015; Li et al., 2016; Xia, 2016; Cao et al., 2017; Rivera-Cárcamo and Serp, 2018). Pd nanoparticles supported on N-CNMs have been used for the oxidation of organic substrates with O_2_ (Arrigo et al., 2012; Ayusheev et al., 2014). The selective oxidation of benzyl alcohol to benzaldehyde was demonstrated (Arrigo et al., 2012). Recently, we have shown that a heterogeneous catalyst based on a divanadium-substituted γ-Keggin phosphotungstate, [γ-PW_10_V_2_O_40_]^5−^ (PW_10_V_2_), and bamboo-like N-CNTs effectively catalyzed the selective oxidation of 2,3,6-trimethylphenol to trimethyl-1,4-benzoquinone with aqueous H_2_O_2_ and could be reused several times without deterioration of the catalytic properties (Evtushok et al., 2018). Catalytic performances of PW_10_V_2_ supported on both CNTs and N-CNTs were also assessed in alkene epoxidation (Evtushok et al., 2019). In the latter case, activity and H_2_O_2_ utilization efficiency were found to be lower relative to homogeneous PW_10_V_2_. The catalysts PW_10_V_2_/N-CNTs were stable under turnover conditions while binding of PW_10_V_2_ to CNTs was not strong enough to avoid POM leaching.

Herein, we explore further the potential of CNTs and N-CNTs as supports for designing selective oxidation catalysts and report immobilization of PW_4_ on N-free and N-doped carbon nanotubes and catalytic performances of these catalysts in H_2_O_2_-based epoxidation of various alkenes. Sulfoxidation of thioethers in the presence of PW_4_/CNTs was also investigated. A dependence of activity and selectivity on the catalyst acidity, which was determined by the preparation procedure, was demonstrated. Catalysts PW_4_/CNTs proved to be highly efficient, truly heterogeneous, and recyclable for both epoxidation and sulfoxidation reactions.

## Experimental

### Materials

Styrene (99%) and methyl phenyl sulfide (99%) were purchased from Acros, 4-bromothioanisole (>98%) and methyl *p-*tolyl sulfide (97%) were obtained from Lancaster, and cyclooctene (98%), cyclohexene (98%), and benzyl phenyl sulfide (>98%) were obtained from Fluka. Acetonitrile (Panreac, HPLC grade) was dried and stored over activated 4-Å molecular sieves. All other reagents and solvents were the best reagent grade and were used without further purification. The concentration of H_2_O_2_ (ca. 30 wt. % in water) was determined iodometrically prior to use.

### Catalysts and Supports

A tetrahexylammonium salt of PW_4_, (THA)_3_[PO_4_{WO(O_2_)_2_}_4_] (THA-PW_4_), was prepared following a previously reported protocol with some modifications (Aubry et al., 1991). To 0.72 g of H_3_PW_12_O_40_ (0.25 mmol), 3 ml of 30% H_2_O_2_ was added. The mixture was stirred until complete dissolution of H_3_PW_12_O_40_. Then, 0.1 ml of 5 M H_3_PO_4_ was added and the solution was stirred for 4 h at 40°C. After that, a solution of THACl (0.88 g in 12 ml of 30% H_2_O_2_) was added to the reaction mixture. The white precipitate was filtered off, washed with water, and dried in air. The purity of the compound was confirmed by IR (characteristic bands: 977, 850, 844, 726, 660, 648, 592, 573, 550, 524 cm^−1^) and ^31^P NMR (δ = 3.8 ppm in MeCN) spectroscopic techniques.

The nitrogen-free and nitrogen-doped carbon nanotubes were synthesized by standard CVD technique described in the previous papers (Suboch et al., 2016; Podyacheva et al., 2017). The as-synthesized CNTs and N-CNTs (1.8 at. % N) were then thoroughly washed in HCl to remove the growth Fe-containing catalyst (Xia, 2016). The acid washing reduced the content of the initial catalyst down to 1–2 wt. %. The remaining catalyst particles were encapsulated within the carbon framework, as evidenced by the inertness of the N-free CNTs in the decomposition of H_2_O_2_ (Evtushok et al., 2018). The washed CNTs and N-CNTs were dried in an Ar flow at 170°C.

Prior to PW_4_ immobilization, CNTs and N-CNTs were preliminarily dried in vacuum at 100°C. Immobilization of PW_4_ was carried out by adsorption from MeCN solutions (C (PW_4_) = 1.5 mM) at room temperature, following the protocol described for PW_10_V_2_ (Evtushok et al., 2019). In some cases, HClO_4_ was added in the amount of 1-3 equiv relative to POM to enhance the adsorption capacity. The completion of the adsorption process was controlled by UV–vis (λ = 250 nm). The resulting solid was separated by filtration, thoroughly washed with MeCN and dried in vacuum at room temperature prior to use in catalytic experiments and physico-chemical measurements. For example, in order to obtain 15 wt. % PW_4_/CNTs catalyst, 200 mg of CNTs was taken and placed in 3 ml of MeCN. Then, 35.5 mg of PW_4_ was added upon stirring, followed by immediate addition of 6 μl of 7 M HClO_4_. The mixture was left stirring for 30 min. The catalyst was separated by filtration, thoroughly washed with MeCN, and dried in vacuum at room temperature. The PW_4_ content in the mother liquor was measured using UV–vis spectroscopy.

### Catalytic Oxidation and Product Analysis

Catalytic experiments were carried out in thermostated glass vessels under vigorous stirring (500 rpm). Typical reaction conditions for alkene oxidation were as follows: substrate 0.1 mmol, H_2_O_2_ 0.1–0.2 mmol, catalyst 0.7 μmol PW_4_, MeCN 1 ml, 50°C. Typical reaction conditions for sulfides oxidation were as follows: substrate 0.1 mmol, H_2_O_2_ 0.1 mmol, catalyst 0.33–1 μmol PW_4_, MeCN 1 ml, 27°C. All reactions were started with the addition of H_2_O_2_. The reaction products were identified by GC-MS and quantified by GC using biphenyl as an internal standard. MPS consumption was determined by HPLC using biphenyl as an internal standard. Aliquots of 1.3 μl of the reaction mixture were taken periodically and diluted with 100 μl of *i*PrOH before analysis. The amount of H_2_O_2_ at the end of the reaction was determined iodometrically. Before reuse, the catalysts were separated by filtration, washed with dimethyl carbonate, and dried in vacuum at 50°C for 4 h. Each experiment was reproduced at least two to three times.

### Instrumentation

GC analyses were performed using a gas chromatograph Chromos GC-1000 equipped with a flame ionization detector and a quartz capillary column BPX5 (30 m × 0.25 mm). HPLC measurements were performed using HPLC Agilent Technologies 1220 Infinity LC using ZORBAX Eclipse Plus C-18 column (4.6 × 150 mm, 5-Micron, H_2_O-*i*PrOH = 40:60, 1 ml/min, 25°C). ^31^P NMR spectra were recorded on a Bruker Avance III 500 spectrometer at 161.67 MHz. The chemical shift for P, δ, was determined relative to 85% H_3_PO_4_. UV-vis spectra were recorded on a Varian Cary 60 UV-vis spectrophotometer. X-ray photoelectron spectra were collected on a KRATOS ES300 photoelectron spectrometer with non-monochromatic AlK_α_ radiation (hν 1486.6 eV). The Au4f_7/2_ and Cu2p_3/2_ core-level lines with binding energies 84.0 eV and 932.7 eV, respectively, were used for the spectrometer calibration. The nitrogen content in N-CNTs was defined as N/C ratio (at. %) from quantitative estimations using XPS data. High-resolution transmission electron microscopy (HRTEM) data were obtained using a JEM-2200FS (JEOL Ltd., Japan) electron microscope operated at 200 kV for obtaining TEM images. Images with a high atomic number contrast were acquired using a high-angle annular dark-field (HAADF) detector in scanning-TEM (STEM) mode. The local composition of the samples was studied using EDX spectroscopy (JEOL Ltd., Japan) with a resolution of 130 eV. The samples for the TEM study were prepared on perforated carbon film mounted on a copper grid. FTIR spectra (4,000–350 cm^−1^, 40 scans, resolution 4 cm^−1^) were obtained in a nujol mull using a Cary 660 FTIR spectrometer (Agilent Technologies). When obtaining the IR spectrum of a bulk PW_4_ sample, a standard sample-Nujol ratio of 3–5 mg per 0.2–0.5 ml was used. To collect the IR spectrum of the supported PW_4_, the ratio was increased by about 5–7 times. Nitrogen adsorption measurements were carried out at 77 K using an ASAP-2400 instrument. The content of tungsten in the filtrate was determined by ICP–OES using a PerkinElmer Optima−430 DV instrument.

## Results and Discussion

### Catalysts Synthesis and Characterization

Adsorption of PW_4_ on both CNTs and N-CNTs was carried out from a MeCN solution. Since our previous studies showed that preliminary drying of N-CNTs is essential to increase the maximal amount of adsorbed POM (Evtushok et al., 2018), the supports were preliminarily dried in vacuum. Given that an increase in the amount of N in N-CNTs from 1.8 to 9 at.% substantially enhanced unproductive H_2_O_2_ decomposition (Evtushok et al., 2018), a sample of N-CNTs with 1.8 at. % of nitrogen was used. Previously, we demonstrated by XPS that treatment with HClO_4_ favors electrostatic attachment of POM on the surface of both N-free and N-doped CNMs, which occurs via anion exchange (the signal of ${\text{ClO}}_{4}^{-}$ appeared in the XPS spectra after the acid treatment and disappeared after immobilization of PW_10_V_2_) (Evtushok et al., 2019). Taking this into account, various amounts of HClO_4_ were added to the MeCN solution during immobilization of PW_4_.

The maximal amount of PW_4_ adsorbed on N-CNTs reached ca. 15 wt. % when 1 equiv of HClO_4_ was added during the preparation. Similarly to PW_10_V_2_ (Evtushok et al., 2018), the adsorption of PW_4_ was fast and proceeded to completion within 15 min. For the CNTs, at least 2 equiv of HClO_4_ was needed to achieve 15 wt. % PW_4_ content in PW_4_/CNTs. Importantly, catalysts PW_4_/CNTs can be prepared without acid, but then the maximum POM content is limited to 5 wt. %. Also, in this case, the adsorption proceeded slowly and took about 24 h.

**Figure 1:**
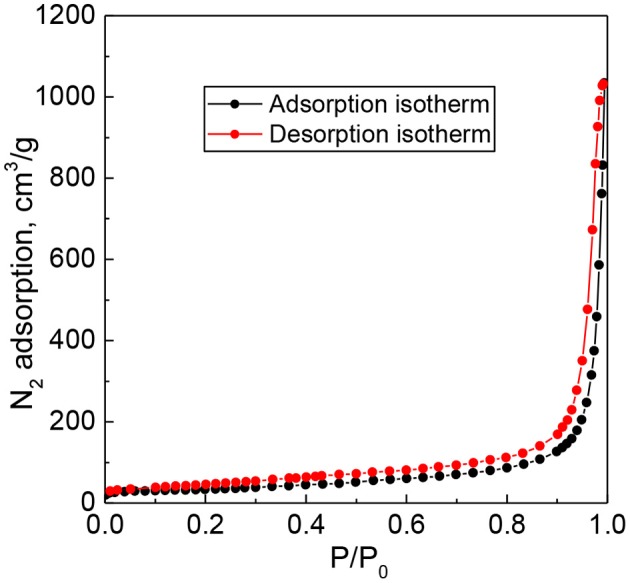
Nitrogen adsorption–desorption isotherms for 15 wt. % PW_4_/CNTs catalyst.

Nitrogen adsorption–desorption isotherms for a representative 15 wt. % PW_4_/CNTs catalyst are presented in Figure 1. The material revealed adsorption isotherms of type IV in the IUPAC classification, typical of mesoporous materials.

Textural characteristics of the supports and immobilized PW_4_ catalysts acquired from the N_2_ adsorption data are presented in Table 1 along with elemental analysis data. Some reduction in the surface area and mesopore volume could be observed for both CNTs and N-CNTs after immobilization of PW_4_.

**Table 1:** Textural properties and elemental analysis data for CNTs, N-CNTs and supported PW_4_.

**Support**	**N^a^ at. %**	**PW_4_ wt. %**	**S_BET_^b^ m^2^/g**	**V_pore_^c^ cm^3^/g**
CNTs	0	-	150	0.70
CNTs	0	5	148	0.64
CNTs	0	15	121	0.41
N-CNTs	1.8	-	170	0.59
N-CNTs	1.8	15	126	0.44

a *N, at.%, corresponds to the N/C ratio according to XPS data*.

b *Surface area*.

c *Mesopore volume*.

**Figure 2:**
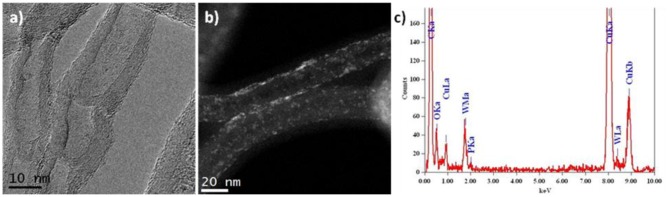
TEM data for 15 wt. % PW_4_/N-CNTs: **(a)** HRTEM, **(b)** HAADF-STEM images, and **(c)** EDX spectrum.

Figure 2a presents a HRTEM image of N-CNTs with bamboo-like packing of graphite layers containing 15 wt. % of PW_4_. Such morphology is typical of nitrogen-doped carbon nanotubes. Images obtained in the HAADF-STEM mode where POM particles give a bright light contrast (Figure 2b) revealed that the particles are distributed over the surface of the nanotubes with some degree of aggregation. The diameter of individual particles is <1 nm, which is consistent with the size of the PW_4_ molecule. The EDX spectrum of 15 wt. % PW_4_/N-CNT gives a ratio of P:W equal to 1:4 (Figure 2c).

**Figure 3:**
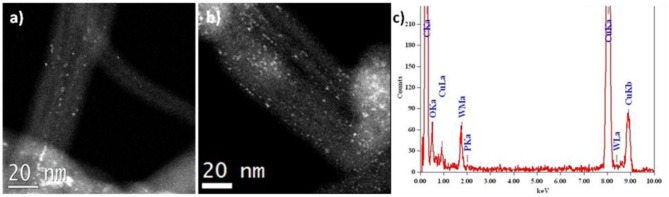
HAADF-STEM images of **(a)** 5 wt. % PW_4_/CNTs prepared without acid and **(b)** 15 wt. % PW_4_/CNTs prepared with 2 equiv HClO_4_; **(c)** EDX spectrum for 15 wt. % PW_4_/CNTs.

Figure 3 shows a comparison of the HAADF-STEM images of two catalysts PW_4_/CNTs with different PW_4_ content and amount of HClO_4_ added during the preparation. For both catalysts, a quasi-molecular dispersion of PW_4_ is observed. The amount of the PW_4_ particles is lower for 5 wt. % PW_4_/CNTs, as expected. The EDX spectrum of 15 wt. % PW_4_/CNT confirmed the ratio of P:W close to 1:4.

Interestingly, an opposite trend was observed for the Keggin polyanion PW_10_V_2_ immobilized on N-doped and N-free CNTs: a better molecular dispersion of the POM species was found for N-CNTs rather than CNTs (Evtushok et al., 2019). Therefore, we may conclude that the nature of POM affects the immobilization mode.

**Figure 4:**
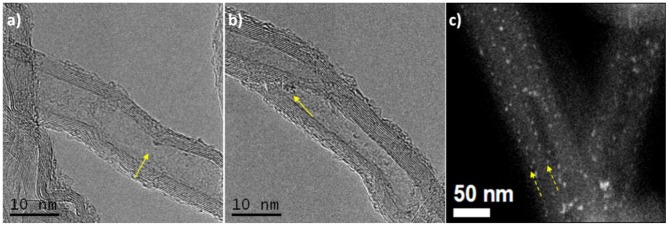
**(a,b)** HRTEM images of 5 wt. % PW_4_/CNTs, **(c)** HAADF-STEM image of 15 wt. % PW_4_/CNTs.

HRTEM images of 5 wt. % PW_4_/CNTs prepared without acid might indicate that PW_4_ molecules are able to enter inside CNTs channels during the adsorption process (Figure 4). A HAADF-STEM image of the 15 wt. % PW_4_/CNTs catalyst (Figure 4c) shows that PW_4_ particles line up along the line both outside the nanotube and along the surface of the inner cavity of the tube. This may evidence that some POM particles penetrate exactly inside the tube (if these particles were located simply on the surface, they would be arranged in a chaotic manner). This might be a possible explanation of the long duration of the adsorption process.

### Catalytic Performance of PW_4_/N-CNTs and PW_4_/CNTs in Alkene Epoxidation

Catalytic performances of PW_4_/N-CNTs and PW_4_/CNTs containing 15 wt. % of the active complex were assessed in cyclooctene (CyO) epoxidation with aqueous H_2_O_2_ in various solvents and compared with the performance of homogeneous THA-PW_4_ (Table 2).

**Table 2:** CyO epoxidation with H_2_O_2_ catalyzed by PW_4_/N-CNTs, PW_4_/CNTs, and THA-PW_4_.

**Entry**	**Catalyst**	**Solvent**	**Time,^a^ h**	**CyO conversion, %**	**Epoxide select,^b^ %**	**TOF,^c^ h^**−1**^**
1	15 wt. % PW_4_/N-CNTs	MeCN	2	95	>99^b^	142
2	15 wt. % PW_4_/N-CNTs	EtOAc	2	43	97	101
3	15 wt. % PW_4_/N-CNTs	DMC	0.85	37	89	136
4	15 wt. % PW_4_/N-CNTs	MeOH	2	28	15	31
5	N-CNTs	MeCN	4	50	0	-
6	15 wt. % PW_4_/CNTs	MeCN	1.5	95	>99^c^	197
7	15 wt. % PW_4_/CNTs	DMC	0.85	93	97	290
8	CNTs	DMC	4	10	0	-
9	THA-PW_4_	MeCN	5	85	96	43
10	THA-PW_4_	DMC	5	80	100	44

*Reaction conditions: [CyO] = 0.1 M (13 μl), [H_2_O_2_] = 0.2 M (20 μl 30% H_2_O_2_), 15 wt. % PW_4_/N-CNTs (or /CNTs) 10 mg (0.7 μmol PW_4_), solvent 1 ml, 50°C*.

a *Time required to achieve maximum conversion*.

b *Epoxide yield based on the substrate consumed*.

c *TOF = (moles of substrate consumed) × (moles of PW_4_)^−1^ × time^−1^. Determined by GC from initial reaction rates*.

The reaction in the presence of PW_4_/N-CNTs in MeCN showed high conversion of 95% and epoxide selectivity close to 100% (Table 2, entry 1). In ethyl acetate and dimethyl carbonate (DMC), much lower conversions (43 and 37%, respectively) could be attained despite similar initial rates of the reaction (Table 2, entries 2 and 3). CyO oxidation over PW_4_/N-CNTs in methanol gave epoxide with negligible selectivity at low substrate conversion (Table 2, entry 4). Moreover, treatment of the catalyst with MeOH resulted in significant leaching of PW_4_, which can be detected by UV-vis spectroscopy and elemental analysis. A blank experiment showed that the reaction of CyO with H_2_O_2_ in the presence of POM-free N-CNTs in MeCN could give a conversion of 50%, but no epoxide was found among the reaction products (Table 2, entry 5). This result agrees with the previously reported results on N-CNT activity in different oxidation reactions due to the presence of nitrogen species (García-Bordejé et al., 2014).

With the PW_4_/CNTs catalyst, high conversions (93–95%) and epoxide selectivities (97–100%) were observed in both acetonitrile and DMC (Table 2, entries 5 and 6); in the latter, the reaction was faster (TOF 290 vs. 197 h^−1^ in MeCN). In contrast to N-CNTs, N-free CNTs were practically inactive in the transformation of CyO (Table 2, entry 8). Epoxidation of CyO in the presence of THA-PW_4_ in MeCN and DMC proceeded with 96–100% selectivity at 80–85% conversion (Table 2, entries 9 and 10). However, activity of the homogeneous catalyst (TOF = 43-44 h^−1^) was much lower than that of the supported catalysts, which is quite unusual for catalysis with PW_4_. This can be rationalized if we remember that acid additives were employed in the preparation of the supported PW_4_ catalysts. Indeed, PW_4_/CNTs (2 equiv of HClO_4_ was used in the synthesis) revealed higher activity in terms of TOF than PW_4_/N-CNTs (1 equiv of HClO_4_). To verify this suggestion, we checked how acid additives affect alkene epoxidation in the presence of THA-PW_4_ (vide infra).

Importantly, the oxidant utilization efficiency of the PW_4_/CNTs catalyst was close to 100%, which is consistent with the extremely low rate of H_2_O_2_ decomposition by the Venturello complex (Duncan et al., 1995) and CNTs (Evtushok et al., 2018). The catalyst PW_4_/N-CNTs demonstrated a lower H_2_O_2_ utilization efficiency (70%) due to the significant decomposition of hydrogen peroxide on N-CNTs (Evtushok et al., 2018).

The results acquired for the CyO epoxidation allowed us to conclude that N-free CNTs are optimal supports for PW_4_ because PW_4_/CNTs catalysts enable the use of the greener solvent DMC and possess very low activity in unproductive H_2_O_2_ decomposition.

**Figure 5:**
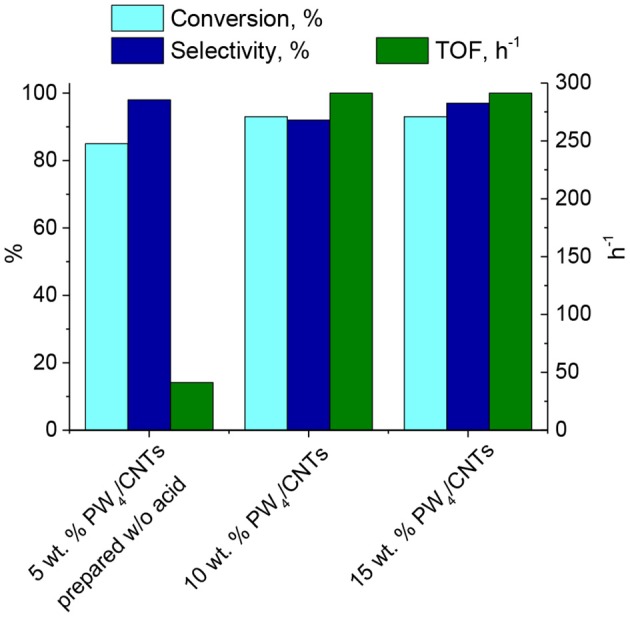
CyO epoxidation over PW_4_/CNTs catalysts with various PW_4_ content. Reaction conditions: [CyO] = 0.1 M (13 μl), [H_2_O_2_] = 0.2 M (20 μl 30% H_2_O_2_), PW_4_/CNTs 10–30 mg (0.7 μmol PW_4_), DMC 1 ml, 50°C.

The amount of PW_4_ adsorbed on CNTs was varied in order to achieve an optimal catalyst composition. Catalytic properties of PW_4_/CNTs catalysts with various PW_4_ content in CyO epoxidation are presented in Figure 5. Note that the addition of acid (2 equiv relative to PW_4_) was required to obtain catalysts containing 10 and 15 wt. % of the active complex.

Substrate conversion and epoxide selectivity in the CyO oxidation over PW_4_/CNTs changed insignificantly with decreasing PW_4_ content from 15–10%. The activity (TOF) also remained constant, which implies a fairly good dispersion and accessibility of the active complex in both samples. The catalyst 5 wt. % PW_4_/CNTs prepared without acid showed a bit lower conversion (85 vs. 93%) and superior selectivity close to 100%. Although activity of this catalyst was significantly lower in comparison to the catalysts prepared with acid (TOF = 41 vs. 290 h^−1^), it was almost identical to that of homogeneous THA-PW_4_ (TOF = 44 h^−1^). This comparison again indicates the rate-accelerated role of acid in the catalytic activity of PW_4_ catalysts, which is in agreement with the literature (Witte et al., 2004).

Oxidation of more challenging alkenes, such as cyclohexene and 3-carene (their epoxides are sensitive to acid-catalyzed hydrolysis; Villa de et al., 1999; Neimann and Neumann, 2000) in the presence of supported PW_4_ catalysts and homogeneous THA-PW_4_ was studied to evaluate the role of acidity for the catalytic performance of PW_4_/CNTs catalysts. The results are summarized in Table 3.

**Table 3:** Oxidation of cyclohexene and 3-carene with H_2_O_2_ in the presence of PW_4_ catalysts.

**Entry**	**Alkene**	**Catalyst**	**HClO_**4**_, equiv^a^**	**Time, min**	**Conv., %**	**Epoxide select.,^b^%**
1	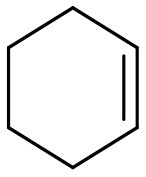	15 wt. % PW_4_/CNTs	2	60	82	2
2		5 wt. % PW_4_/CNTs	0.2	240	66	79
3		5 wt. % PW_4_/CNTs	0	240	46	83
4	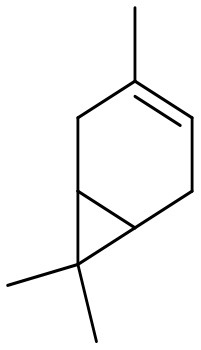	15 wt. % PW_4_/CNTs	2	90	55	2
5		5 wt. % PW_4_/CNTs	0.2	240	50	15
6		5 wt. % PW_4_/CNTs	0	300	50	80
7		THA-PW_4_	1	90	78	7
8		THA-PW_4_	0	300	42	99

*Reaction conditions: [Alkene] = 0.1 M, [H_2_O_2_] = 0.1 M (10 μl 30% H_2_O_2_), PW_4_/CNTs 10–30 mg (0.7 μmol PW_4_), DMC 1 ml, 50°C*.

a *Amount of HClO_4_ (equiv to PW_4_) added during preparation of supported catalysts*.

b *GC yields based on substrate consumed. Another product of cyclohexene oxidation was 1,2-cyclohexanediol. Oxidation of 3-carene yielded also 3,4-caranediol, p-cymene, cis- and trans-caranones*.

The catalyst 15 wt. % PW_4_/CNTs (during its preparation, 2 equiv of HClO_4_ with respect to PW_4_ was added) showed negligible selectivity (~2%) to epoxides for both cyclohexene and 3-carene (Table 3, entries 1 and 4). For cyclohexene, the main oxidation product was the epoxide-ring opening product 1,2-cyclohexanediol while 3-carene gave diol, *p*-cymene along with *cis*- and *trans*-caranones. The catalyst prepared with a lower amount of acid, 5 wt. % PW_4_/CNTs (0.2 equiv of HClO_4_ to PW_4_), revealed fairly good selectivity for epoxide formation in the oxidation of cyclohexene (79%) but poor selectivity (only 15%) with 3-carene (Table 3, entries 2 and 5), indicating that the optimal synthesis protocol depends on the specific olefin. The highest selectivity toward epoxides was achieved in the presence of the 5 wt. % PW_4_/CNTs catalyst that was prepared without any acid (Table 3, entries 3 and 6). Similar trends were observed with homogeneous THA-PW_4_ catalyst (Table 3, entries 7 and 8). Importantly, overall selectivity for epoxides and ring-opening products was >90% in all the reactions and only 1–2% of allylic oxidation products formed, indicating heterolytic activation of the oxidant.

Thus, selectivity of all PW_4_ catalysts decreased significantly with increasing acidity if acid-sensitive substrates/products were employed/produced. When considering the activity of these catalysts, it becomes clear that it follows the opposite trend. Given that, a careful balance between activity and selectivity should be maintained. It is noteworthy that the composition (preparation) of catalysts PW_4_/CNTs can be readily tuned to achieve an optimal performance in epoxidation of various olefins.

### Catalytic Performances of PW_4_/CNTs Catalysts in Selective Oxidation of Sulfides

**Scheme 1:**
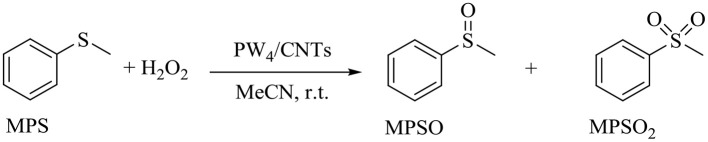
Oxidation of MPS with H_2_O_2_ in the presence of PW_4_/CNTs.

The catalytic performance of PW_4_/CNTs catalysts was also assessed in the selective oxidation of organic sulfides with H_2_O_2_ using methyl phenyl sulfide (MPS) as a model substrate (Scheme 1) and compared with that of homogeneous PW_4_.

First of all, the effect of solvent nature on the MPS oxidation over PW_4_ catalysts was studied. The main results are collected in Table 4.

**Table 4:** Catalytic oxidation of MPS with H_2_O_2_ over CNTs, THA-PW_4_, and PW_4_/CNTs.

**Entry**	**Catalyst (μmol PW_**4**_)**	**Solvent**	**Time, h**	**Sulfide conversion, %**	**Sulfoxide sel., %**	**Sulfone sel., %**
1	-	MeCN	24	14	79	21
2	CNTs	MeCN	24	16	69	31
3	THA-PW_4_ (1)	MeCN	0.25	93	83	17
4	THA-PW_4_ (1)	DMC	0.25	86	70	30
5^a^	THA-PW_4_ (1)	MeCN	0.5	92	86	14
6	15 wt. % PW_4_/CNTs (1)	MeCN	2.5	93	90	10
7	15 wt. % PW_4_/CNTs (1)	DMC	2.5	86	80	20
8	15 wt. % PW_4_/CNTs (0.3)	MeCN	6	93	92	8
9	5 wt. % PW_4_/CNTs (0.3)^b^	MeCN	3	88	84	16

*Reaction conditions: [MPS] = 0.1 M (11.7 μl), [H_2_O_2_] = 0.1 M (10 μl 30% H_2_O_2_), PW_4_/CNTs 5-15 mg (0.3-1 μmol PW_4_), solvent 1 ml, 27°C*.

a *1 μmol of HClO_4_ was added*.

b *The catalyst was prepared without HClO_4_*.

Blank experiments (Table 4, entries 1 and 2) demonstrated that oxidation of MPS without any catalyst or in the presence of CNTs proceeded slowly and gave a low conversion (ca. 15% after 24 h). Reactions carried out in MeCN and DMC in the presence of homogeneous THA-PW_4_ showed that higher sulfide conversion (93 vs. 86%) and selectivity to sulfoxide (83 vs. 70%) could be achieved using MeCN as solvent (Table 4, entries 3 and 4). The addition of HClO_4_ had insignificant effect on the attainable conversion and product selectivity but slowed the reaction (Table 4, compare entries 3 and 5). In the presence of the heterogeneous catalyst 15 wt. % PW_4_/CNTs, conversion and sulfoxide selectivity were also higher in MeCN (Table 4, entries 6 and 7). Reduction in the catalyst loading did not affect conversion and selectivity of sulfoxidation but it increased the reaction time (Table 2, compare entries 6 and 8).

Then, the effect of catalyst acidity on its activity and selectivity was investigated in MeCN using similar catalyst loadings (Table 4, entries 8 and 9). The selectivity to sulfoxide achieved 92% with 15 wt. % PW_4_/CNTs prepared with 2 equiv of HClO_4_, while catalyst 5 wt. % PW_4_/CNTs, which was prepared without acid, showed lower sulfoxide selectivity (84%). Therefore, catalyst acidity favors the formation of sulfoxide, most likely due to increasing electrophilicity of the oxidizing species (Zalomaeva et al., 2019). However, an unexpected trend was observed for catalyst activity. In the presence of more acidic 15 wt. % PW_4_/CNTs, the oxidation process completed within 6 h, whereas with the PW_4_/CNTs catalyst, the reaction was faster and reached the maximum conversion after 3 h. A similar effect of acid on the reaction rate was observed for homogeneous PW_4_ (Table 4, entries 3 and 5).

With a correction on the active oxygen initially present in PW_4_, the oxidant utilization efficiency was close to 100% for all the catalysts tested in the selective oxidation of MPS.

### Substrate Scope

Table 5 demonstrates the substrate scope in the oxidation of alkenes and sulfides over heterogeneous catalysts PW_4_/CNTs. Catalysts PW_4_/CNTs ensure high or fairly good selectivity of epoxidation for various types of alkenes. However, in all the reactions, complete conversion was not attained with 1 equiv of the oxidant despite high H_2_O_2_ utilization efficiency. Duncan et al. showed that PW_4_ loses its activity because of the reaction with epoxidation products during a prolonged stay (Duncan et al., 1995). Catalytic epoxidation of cyclooctene over PW_4_/CNTs in the presence of 0.1 M 1,2-cyclooctanediol or cyclooctene oxide showed no signs of a decrease in the reaction rate or any decrease in conversion. Recycling experiments demonstrated that the observed catalyst deactivation is reversible and PW_4_/CNTs catalysts can be successfully regenerated and reused (*vide infra*).

**Table 5:** Catalytic oxidation of various alkenes and sulfides with H_2_O_2_.

**Substrate**	**Catalyst**	**Solvent**	**HClO_**4**_ added, equiv**	**Time, h**	**Conversion, %**	**Selectivity, %**
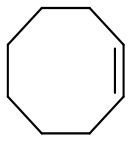	15 wt. % PW_4_/CNTs	DMC	2	2	93	97
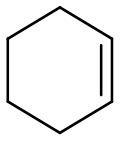	5 wt. % PW_4_/CNTs	DMC	0.2	4	66	79
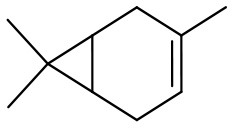	5 wt. % PW_4_/CNTs	DMC	0	5	50	80
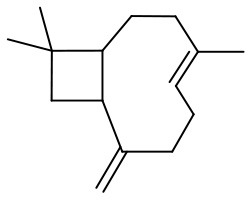	15 wt. % PW_4_/CNTs	DMC	2	1	100	65
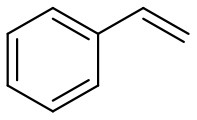	5 wt. % PW_4_/CNTs	DMC	0	7	14	95
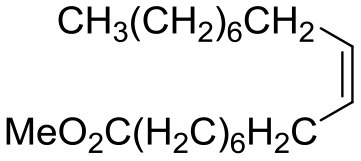	5 wt. % PW_4_/CNTs	DMC	0	4	85	85
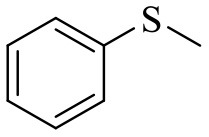	15 wt. % PW_4_/CNTs	MeCN	2	2.5	93	90
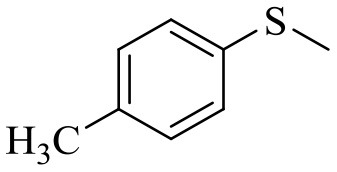	15 wt. % PW_4_/CNTs	MeCN	2	2	90	88
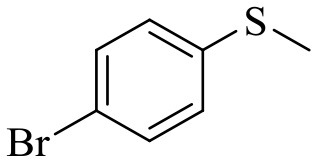	15 wt. % PW_4_/CNTs	MeCN	2	2	94	89
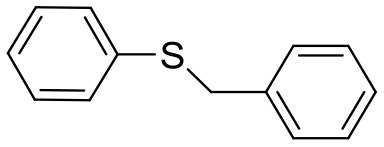	15 wt. % PW_4_/CNTs	MeCN	2	2	86	81

*Reaction conditions: [Substrate] = 0.1 M, [H_2_O_2_] = 0.1 M (10 μl 30% H_2_O_2_), solvent 1 ml, 50°C (for alkenes), or 27°C (for sulfides). Catalyst 10–30 mg (0.7 μmol PW_4_ for alkenes) or 15 mg (1 μmol PW_4_ for sulfides)*.

It is a matter of common observation that selectivity of epoxidation in the presence of either homogeneous or heterogeneous catalysts is affected by the catalyst acidity and concentration of H_2_O in the reaction mixture (Herrmann et al., 1994; Hutchings et al., 1996; Hutter et al., 1999). Biphasic catalytic systems based on PW_4_ exhibit excellent selectivity for acid-sensitive epoxides as the water concentration is very low in the hydrophobic organic phase (Venturello et al., 1983, 1985; Ishii et al., 1988; Sakaguchi et al., 1996). Homogeneous catalytic systems based on THA-salt of PW_4_ are less selective due to inherent Lewis acidity of PW_4_ (Kamata et al., 2014) and the presence of water in the reaction mixture, which is added along with the peroxide. Since selectivity, conversion, and activity achieved in epoxidations over 5 wt. % PW_4_/CNTs are very close to those acquired for homogeneous systems based on THA-salt of PW_4_ (Swalus et al., 2013; Kamata et al., 2014), we may suppose that the catalytic performance of the PW_4_/CNTs catalysts in alkene epoxidation can be further improved by hydrophobization of the CNTs surface.

Catalyst PW_4_/CNTs was also active and revealed high selectivity to sulfoxide in the oxidation of sulfides with various *p*-substituents as well as benzyl phenyl sulfide. For the latter, the sulfide conversion and selectivity to sulfoxide were slightly lower in comparison with other sulfides studied.

### Catalyst Stability and Reusability

**Figure 6:**
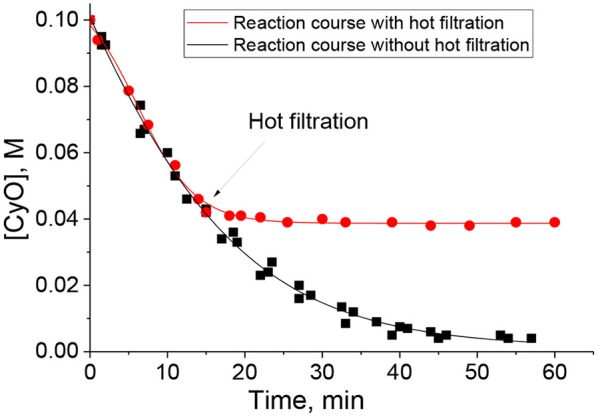
Hot catalyst filtration test for CyO oxidation with H_2_O_2_ over 15 wt. % PW_4_/CNTs in DMC. Reaction conditions: [CyO] = 0.1 M (13 μl), [H_2_O_2_] = 0.2 M (20 μl 30% H_2_O_2_), 15 wt. % PW_4_/CNTs 10 mg (0.7 μmol PW_4_), DMC 1 ml, 50°C.

The elemental analysis revealed minor leaching of tungsten (5 ppm) during the catalytic CyO oxidation in the presence of PW_4_/N-CNTs in MeCN. On the other hand, a hot filtration test implemented for 15 wt. % PW_4_/CNTs-catalyzed epoxidation in DMC proved the truly heterogeneous nature of the catalysis (Figure 6) and, in this case, the elemental analysis showed no sign of tungsten leaching (<1 ppm). Elemental analysis of the reaction mixture after separation of the catalyst 5 wt. % PW_4_/CNTs also revealed less than 1 ppm of W. A hot filtration test confirmed the heterogeneous nature of the catalysis in sulfoxidation reaction over 15 wt. % PW_4_/CNTs.

**Figure 7:**
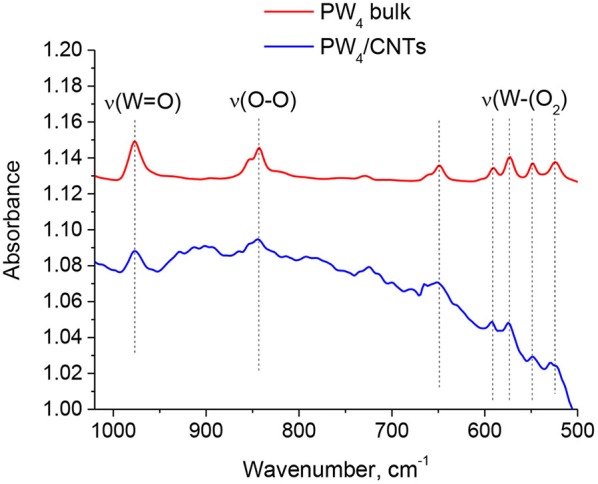
FTIR spectra of bulk PW_4_ and 15 wt. % PW_4_/CNTs catalyst recovered from the reaction mixture (the spectrum of CNTs was subtracted).

FTIR spectra of bulk PW_4_ and 15 wt. % PW_4_/CNTs recovered from the reaction mixture are presented in Figure 7. The main absorption bands characteristic of PW_4_ can be easily distinguished in the subtraction spectrum of the PW_4_/CNTs catalyst, which confirms preservation of the Venturello structure during the catalytic reaction.

**Figure 8:**
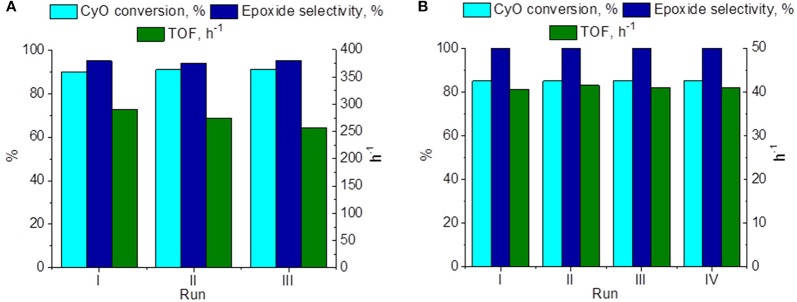
Recycling performance of **(A)** 15 wt. % PW_4_/CNTs and **(B)** 5 wt. % PW_4_/CNTs in CyO epoxidation. Reaction conditions: [CyO] = 0.1 M (13 μl), [H_2_O_2_] = 0.2 M (20 μl 30% H_2_O_2_), PW_4_/CNTs 10-30 mg (0.7 μmol PW_4_), DMC 1 ml, 50°C.

Reusability of the catalyst 15 wt. % PW_4_/CNTs was studied in several consecutive operation cycles of CyO epoxidation (Figure 8A). Neither substrate conversion nor epoxide selectivity changed significantly during the recycling, although some progressive loss in activity expressed in TOF values could be observed. On the other hand, the catalyst 5 wt. % PW_4_/CNTs, which was prepared without acid, turned out to be fully recyclable during, at least, four catalyst reuses (Figure 8B). Between cycles, the catalyst was washed with DMC and then evacuated at 50°C for 4 h.

**Figure 9:**
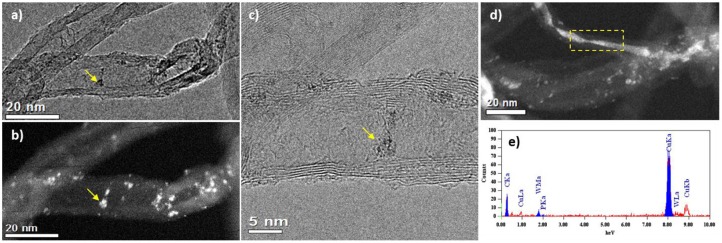
TEM **(a)** and HAADF-STEM **(b)** images of the same region of 15 wt. % PW_4_/CNTs catalyst after reaction; **(c)** HRTEM image of this area showing agglomerate of PW_4_ particles with the size of 3 nm; **(d)** HAADF-STEM image of a nanotube having inner cavity filled with PW_4_ nanoparticles; **(e)** EDX-spectrum from the region marked in **(d)**.

Although the catalyst 15 wt. % PW_4_/CNTs is more active than 5 wt. % PW_4_/CNTs, the latter certainly has higher stability in terms of TOF. As was mentioned above, the reason for the higher activity of the former is its activity due to use of HClO_4_ in the synthesis. Since no leaching of PW_4_ was observed under the turnover conditions, it is reasonable to assume that the decreasing activity of the catalyst 15 wt. % PW_4_/CNTs might be explained by a gradual loss of the catalyst acidity during the recycling. Another reason could be further penetration of PW_4_ inside the CNTs inner channels leading to lower accessibility of the active component. TEM investigation of the 15 wt. % PW_4_/CNTs catalyst after reaction has shown that under reaction conditions, part of the PW_4_ nanoparticles can move over the nanotube surface forming local agglomerates (Figures 9a–c). We assume that most of these agglomerates are located inside the inner cavities of the CNTs, as we observe them being connected to the inner defects of the tube (Figure 9c). In rare cases, the tube cavity is almost filled on a definite length; the EDX analysis of such area confirms the presence of intense W signal (Figures 9d,e). Such rearrangement of the active component can lead to partial loss of activity of the 15 wt. % PW_4_/CNTs catalyst.

**Figure 10:**
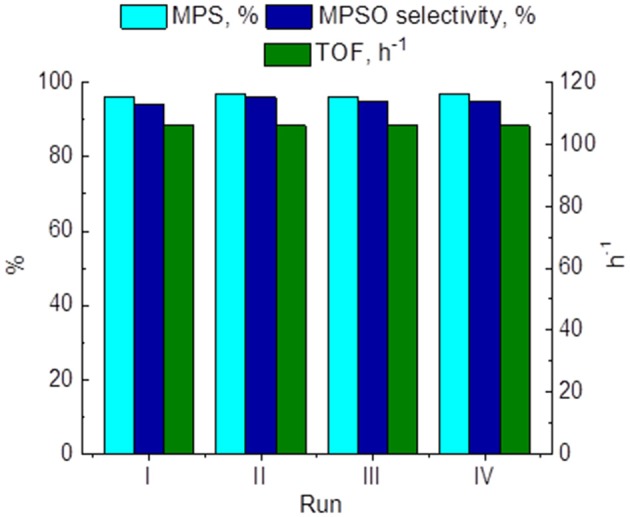
Recycling performance of 15 wt. % PW_4_/CNTs in MPS oxidation. Reaction conditions: [MPS] = 0.1 M (11.7 μl), [H_2_O_2_] = 0.1 M (10 μl), 15 wt. % PW_4_/CNTs, 15 mg (1 μmol PW_4_), MeCN 1 ml, 27°C.

On the other hand, recycling experiments with 15 wt. % PW_4_/CNTs in MPS oxidation showed retention of the attainable sulfide conversion, sulfoxide selectivity, and activity during at least four reuses (Figure 10). However, washing with DMC between operation cycles was required to preserve the catalyst activity. When the catalyst was washed with MeCN, its activity dropped, but it could be restored to the initial level after additional washing with DMC.

## Conclusions

The Venturello complex [PW_4_O_24_]^3−^ was successfully immobilized on N-CNTs and CNTs. In contrast to the immobilization of the Keggin polyanion [γ-PW_10_V_2_]^5−^, N-doping and additives of HClO_4_ were not obligatory to ensure strong binding and quasi-molecular dispersion of PW_4_ on the surface of CNTs, most likely because of the smaller size of the Venturello complex. N-free CNTs are more preferable than N-CNTs as a support because the former are inert in H_2_O_2_ unproductive degradation and ensure high H_2_O_2_ utilization efficiency. The addition of acid during the immobilization allows one to increase the PW_4_ content in the catalyst. The resulting catalyst acidity affects both catalytic activity and product selectivity. In alkene oxidation, acidity is detrimental for epoxide selectivity while the opposite trend is observed in thioether sulfoxidation. A balance between activity and selectivity can be tuned by a careful control of the amount of acid added during the immobilization of PW_4_. The catalyst 15 wt. % PW_4_/CNTs prepared using 2 equiv of HClO_4_ proved to be highly efficient and truly heterogeneous for the selective epoxidation of cyclooctene and sulfoxidation of various thioethers. However, its activity gradually decreased during recycling, most likely due to the loss of acidity and penetration of PW_4_ inside the CNT inner channels. On the contrary, the catalyst 5 wt. % PW_4_/CNTs prepared without acid demonstrated catalytic properties analogous to homogeneous THA-PW_4_ in the epoxidation of various alkenes and did not lose its activity under the turnover conditions.

## Data Availability Statement

The datasets generated for this study are available on request to the corresponding author.

## Author Contributions

OK contributed conception and design of the study. VE carried out the preparation of catalysts. II performed catalytic oxidation of alkenes. OP and AS synthesized carbon nanomaterials. OS acquired HRTEM and HAADF-STEM images. YC measured IR-spectra. OZ performed catalytic oxidation of thioethers. VE wrote the first draft of the manuscript. OK, VE, II, OZ, OP, and OS wrote sections of the manuscript. All authors contributed to manuscript revision, read, and approved the submitted version.

### Conflict of Interest

The authors declare that the research was conducted in the absence of any commercial or financial relationships that could be construed as a potential conflict of interest.
